# Subjective Experiences and Sensitivities in Women with Fibromyalgia: A Quantitative and Comparative Study

**DOI:** 10.1155/2018/8269564

**Published:** 2018-04-01

**Authors:** P. De Roa, P. Paris, J. L. Poindessous, O. Maillet, A. Héron

**Affiliations:** ^1^Pain Unit, Dreux Hospital, GHT28, France; ^2^Department of Mental Health, Dreux Hospital, GHT28, France; ^3^Center of Treatment and Pain Evaluation, Ambroise Paré Hospital, Paris, France; ^4^Clinical Research Unit URC28, Dreux Hospital, GHT28, France; ^5^Department of Human Physiology, Paris Descartes University, Paris, France

## Abstract

Fibromyalgia is a chronic widespread pain syndrome associated with chronic fatigue. Its pathogenesis is not clearly understood. This study presents subjective experiences and sensitivities reported by fibromyalgia patients, which should be considered in primary care to avoid medical nomadism, as well as stigmatization of the patients. The prevalence of significant characteristics was compared with others patients consulting at the same pain unit who suffer from rebel and disabling form of chronic migraine. Psychometric tests were anonymously completed by 78 patients of the Pain Unit (44 fibromyalgia patients and 34 migraine patients). Tests evaluated pain (Visual Analog scale), childhood traumas (Childhood Trauma Questionnaire), lack of parental affection, stressful life events (Holmes and Rahe Scale), anxiety and depression (Hospital Anxiety and Depression Scale), perceived hypersensitivity to 10 stimuli, and hyperactivity before illness. However, pain scores were comparable in the two groups, and the prevalence was significantly higher in fibromyalgia patients than in migraine patients for anxiety (81.8% versus 51.5%) and depression (57.1% versus 8.8%). Childhood physical abuses were more frequently reported in fibromyalgia than in migraine cases (25% versus 3%). Similarly, the feeling of lack of parental affection, subjective hypersensitivity to stress and stimuli (cold, moisture, heat, full moon, and flavors) or hyperactivity (ergomania), appeared as prominent features of fibromyalgia patients. Fibromyalgia patients considered themselves as being hypersensitive (mentally and physically) compared to migraine patients. They also have higher depression levels. Beyond somatic symptoms, precociously taking account of psychosocial and behavioral strategies would highly improve treatment efficiency of the fibromyalgia syndrome.

## 1. Introduction

Fibromyalgia is a chronic widespread pain syndrome associated with chronic fatigue. It affects 2–4% of the adult population, with a higher incidence in women [1, 2]. Considering the musculoskeletal pain symptoms, the World Health Organization quoted fibromyalgia as a rheumatologic disease (M79.7). If the reality of fibromyalgia syndrome is recognized, at least in its severe form, its causes and pathophysiology remain poorly understood and controversial. In 1990, the American College of Rheumatology specified diagnostic criteria of fibromyalgia [3]. In 2010, new criteria appeared taking into account nonrestorative sleep, cognitive impairment, and variable somatic symptoms associated with chronic pain [4].

Recent studies reported that interaction between genetic predispositions [5, 6], biochemical factors, psychological profiles [7], and triggering events which sensitize the central nervous system [8–10], could contribute to the etiology of the fibromyalgia syndrome.

During the clinical examination of painful patients at the Chronic Pain Unit, we used a semistructured interview. The consultation usually lasted 90 minutes and focused on the life history. The attentive listening of fibromyalgia patients revealed life adversity, especially during childhood. Patients usually reported lack of affection, indifference, neglect, or abuse from their family, in accordance with a recent meta-analysis which suggested that childhood traumas could be associated with fibromyalgia syndrome [11, 12]. In addition, patients often mentioned other life's traumas: bereavements, abandon, rapes, severe illness, or accidents. They also reported high sensitivity to stimuli and professional harassment.

The present cross-sectional study aimed to characterize childhood experiences, perceived lack of parental affection, hypersensitivity to stimuli, life stressors, anxio-depression, and ergomania mentioned by French fibromyalgia patients. The prevalence of these parameters was quantified using self-report questionnaires and was compared to that assessed in migraine patients treated in the same Pain Unit (as a control group). Indeed, fibromyalgia and migraine both preferentially affected women and resulted in a comparable pain score on the Visual Analogic Scale. They both represented chronic, rebels, and disabling forms of the pathologies, justifying the orientation of the patients to the Pain Unit [4, 13, 14].

## 2. Methods

### 2.1. Study Population

Subjects included in the study were adult women who consulted for fibromyalgia or migraine at the Pain Unit of Dreux Hospital. These patients were generally addressed by a neurologist or a rheumatologist, most often at the tertiary level after medical nomadism. Migraine patients were sent to the Pain Unit because of their resistance to treatments and daily chronic headaches.

In the case of fibromyalgia, the diagnosis was confirmed in the Pain Unit using the criteria of the American College of Rheumatology from 1990 to 2010 in case of fibromyalgia [3, 4]. The pain was chronic, that is, present for more than 3 months and resistant to usual drugs. It was associated with abnormal tenderness, fatigue, stiffness, sleep disturbance, depression, anxiety, and cognitive impairment. The presence of widespread chronic pain was reported on at least 7 of 19 possible tender points of the body and associated with 4 groups of symptoms whose patient quoted the discomfort from 0 to 3 (sum ≥ 6/12): chronic fatigue (more than 3 months), sleep disorders, cognitive disorders, and functional disorders. Additional examinations were normal.

In the case of migraine, the diagnosis had used the criteria of the International Headache Society [13] in case of migraine diagnosis mentioned at least 5 headache attacks lasting 4–72 hours (untreated or unsuccessfully treated). Headache had at least two of the following characteristics: unilateral location, pulsating quality, moderate or severe pain intensity, and aggravation by or causing avoidance of routine physical activity (e.g., walking or climbing stairs), and during headache at least one of the following had occurred: nausea and/or vomiting, photophobia, and phonophobia, not attributed to another disorder. Additional clinical examinations were normal out of crises.

Patients with serious organic pathology evidenced by biological or imagery analysis (inflammatory arthritis and thyroid pathologies), sleep apnea, psychosis, or delirium, were excluded from the study. Fibromyalgia patients with migraine were also excluded from the study to avoid intergroup interferences that may reduce the visibility of the effects.

### 2.2. Evaluation Tools

A set of 6 self-report questionnaires were sent by post to the patients. Questionnaires should be returned anonymously completed, within two months. The set of questionnaires contained the following:

- The Visual Analogue Scale (VAS) to assess subjective perception of global pain on a 10 cm line (0, *no pain*, to 10, *pain as bad it could be*) [15].
- The Childhood Trauma Questionnaire (CTQ) (French version), a 70-item self-administered inventory providing reliable and valid retrospective assessment of child abuse and neglect [16, 17]. Items asked about experiences in childhood and adolescence and were rated on a 5 point Likert-type scale with response options ranging from *Never True* to *Very Often True*. The CTQ had five clinical scales measuring physical and psychological maltreatments: physical, sexual, and emotional abuse, and physical and emotional neglect.
- The Hospital Anxiety and Depression Scale (HADS), determining a score of anxiety and a score of depression [18]. These two scores varied from 0 to 21.
- The Holmes and Rahe stress scale, measuring the level of stress associated with 43 life events that could contribute to illness if occurring in the past 2 years [19]. Events were scored from 11 to 100. If global score >150, the stress level is high or very high (if 150 < total score ≤ 300) with a risk of illness. When total score ≤150, the stress was considered as moderate with a slight risk of illness.
- One questionnaire concerned the sensitivity of the patient to ten different stimuli (light, noise, cold, warm, humidity, flavors, odors, full moon, allergies, and drugs). It allowed evaluation of the subjective sensitivity perceived by the patients. The question was, *“Would you say that you are very sensitive to the following stimuli?”* Possible answers for each stimulus were “*yes”* or “*no*.”
- Three further questions were added two regarding the lack of affection perceived during childhood (*“I have missed affection from my mother/father”*) and another one evaluating the subjective activity level before illness (*“Before my illness, I was a very active person”*). The possible answers were *“Never true,” “Rarely true,” “Sometimes true,” “Often true,”* and *“Very often true,”* respectively, quoted from 1 to 5.

After reception of the filled questionnaires, data analysis was realized on Excel by the Clinical Research Unit. The results were expressed in average ± standard deviation for scores or in percentage for frequencies. The statistic tests comparing fibromyalgia to migraine patients were realized with Student's *t*-test or the test of *χ*_2_ of Pearson. A value of *p* < 0.05 was considered as statistically significant.

## 3. Results

The analysis focused on 78 questionnaires returned by the patients to the Clinical Research Unit (44 from fibromyalgia patients F and 34 from migraine patients M). Main characteristics of the two groups of women were comparable with mean age of 45 ± 12 years and mean pathology duration of 12 ± 10 years.

The Visual Analogic Scores (VASs) evaluating pain during the best moments, the worst moments, and at present (i.e., when patients completed the questionnaire). In the fibromyalgia group, mean VAS varied from 3.3 ± 1.9 during the best moments to 8.9 ± 1.4 during the worst moments. Scores were comparable and not statistically significant in the migraine group (resp. 1.8 ± 2.3 and 8.7 ± 1.2, data not shown).

**Figure 1:**
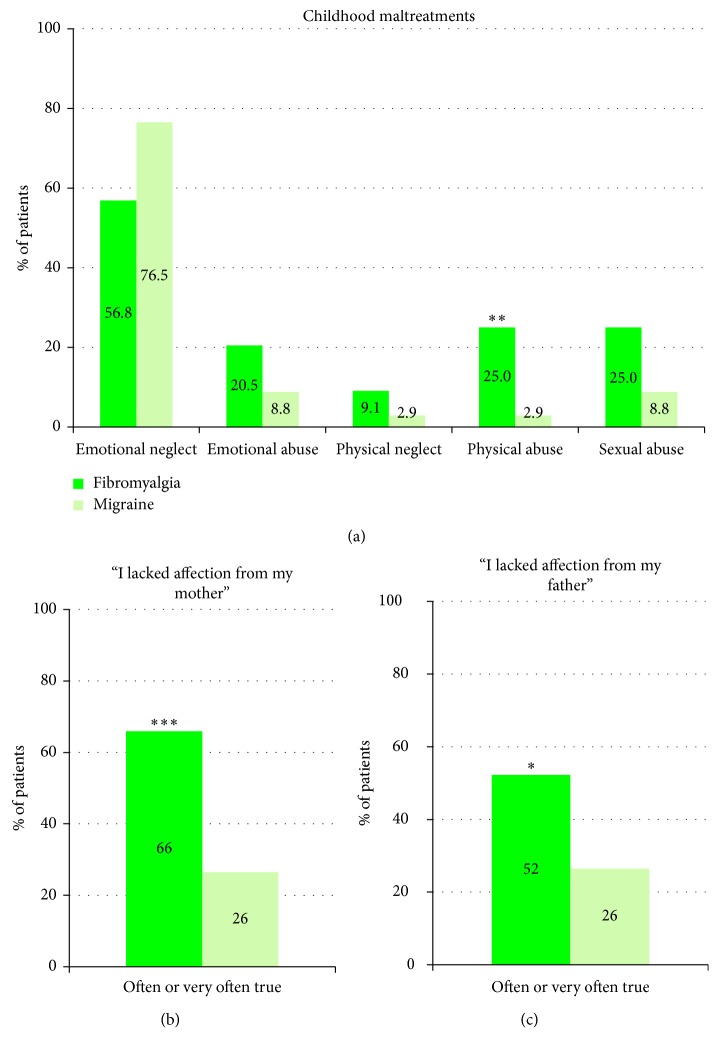
Childhood trauma evaluation. (a) Results of the Childhood Trauma Questionnaire (CTQ) assessing maltreatments of children from their familial environment: percentage of adult fibromyalgia and migraine patients who reported childhood abuses and neglects. (b, c) Percentage of patients who declared having often or very often suffered from maternal (b) or paternal (c) affective deprivation during childhood. Statistical significances between the two groups: ∗*p* < 0.05, ∗∗*p* < 0.01, and  ∗∗∗*p* < 0.001.

Concerning maltreatments retrospectively evaluated by the Childhood Trauma Questionnaire (CTQ), emotional neglect was the most frequently maltreatment reported by fibromyalgia patients (56.8%), followed by physical and sexual abuses (25% of patients), emotional abuse (20.5%), and physical neglect (9.1%) (Figure 1(a)). History of physical abuse was more frequently reported in the fibromyalgia group (25%) than in the migraine group (2.9%) (*p* < 0.01). Physical abuse was defined as bodily assaults on a child by an older person that could lead to or had resulted in injuries. The percentage of patients reporting other maltreatments, emotional maltreatments (humiliation, deamination, and failure of caretakers to provide emotional and psychological needs), physical neglects, or sexual abuses, did not statistically differ between the two groups. However, 52–66% of fibromyalgia patients reported lacks of parental affection versus 26% of migraine patients (*p* < 0.001 for lacks of maternal affection and *p* < 0.05 for lacks of paternal affection) (Figures 1(b) and 1(c)).

**Figure 2:**
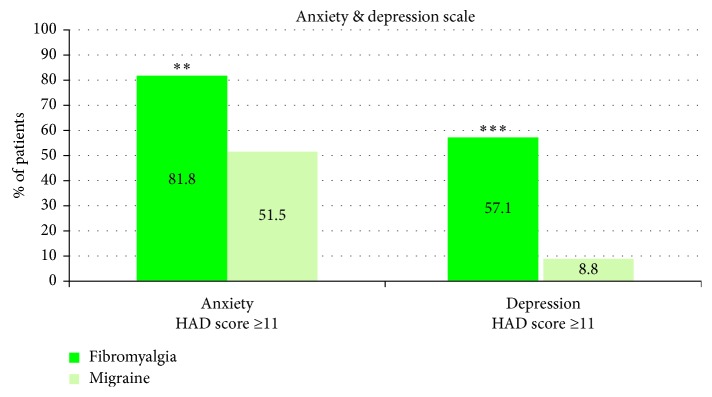
Results of the Hospital Anxiety and Depression (HAD) scale (scores varied from 0 to 21 and score ≥11 was considered as pathological). Percentage of fibromyalgia and migraine patients with pathological anxiety and/or depression. Statistical significances between the two groups: ^∗∗^*p* < 0.01  and  ^∗∗∗^*p* < 0.001.

Prevalence of anxiety and depression was higher in fibromyalgia than in migraine (81.8% versus 51.5%, *p* < 0.01 and 57.1% versus 8.8%, *p* < 0.001, resp.) (Figure 2).

**Figure 3:**
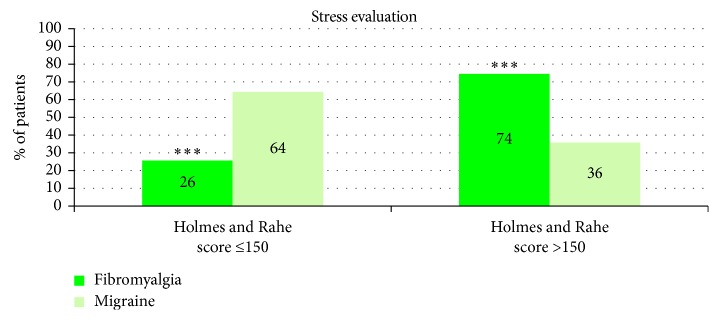
Results of the Holmes and Rahe stress scale measuring the level of stress associated with life events occurred in the past 2 years. Events were scored from 11 to 100. If global score>150, the stress level is high or very high and could contribute to the illness. When total score ≤150, the stress was considered as moderate to low. Results show the percentage of fibromyalgia and migraine patients with low (on the left) or high (on the right) score of stress. Statistical significances between the two groups ^∗∗∗^*p* < 0.001.

Stress evaluation by the Holmes and Rahe test showed that 74% of fibromyalgia patients (versus 36% for migraine patients, *p* < 0.001) reported major life stressors (score >150) to have occurred during the 2 years preceding the test (Figure 3).

**Figure 4:**
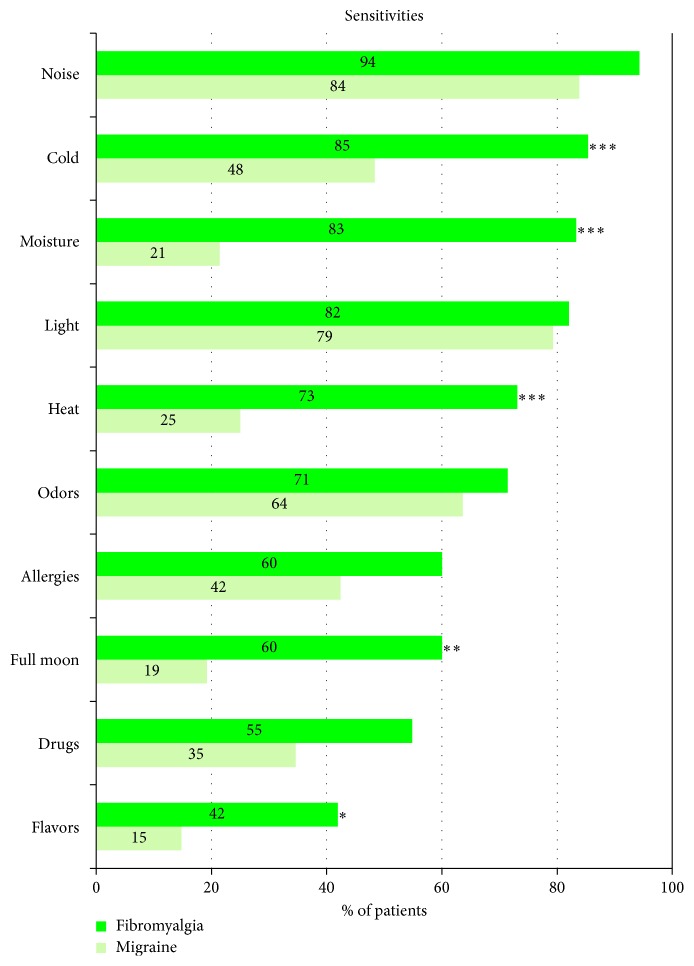
Evaluation of the subjective hypersensitivity perceived by the patients. Questionnaire concerned sensitivity to 10 different stimuli. Results show percentage of fibromyalgia and migraine patients who answered “yes” to the question, “would you say that you are very sensitive to the following stimuli?” Statistical significances between the two groups ^∗^*p* < 0.05, ^∗∗^*p* < 0.01, and  ^∗∗∗^*p* < 0.001.

In addition to stress sensitivity, fibromyalgia patients reported being particularly sensitive to external stimuli, with a significant difference for 5 of them in comparison with migraine patients cold, moisture, heat, full moon, and flavors (Figure 4). The difference was not statistically significant for noise, light, odors, and drugs sensitivity.

**Figure 5:**
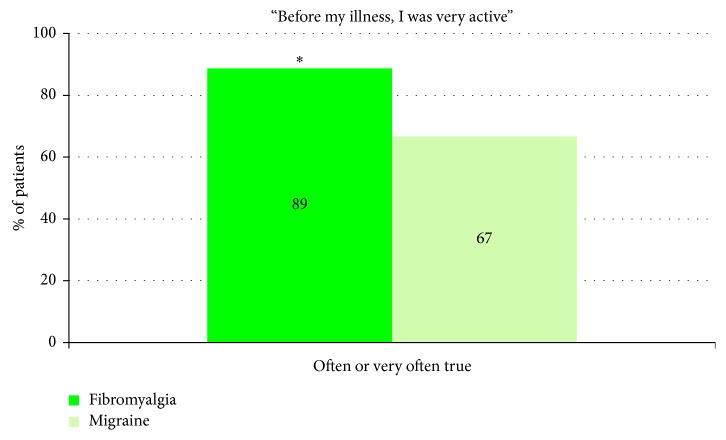
Evaluation of the subjective hyperactivity reported by the patients. Results show percentage of fibromyalgia and migraine patients who answered “Often true” or “Very often true” to the question, “Before my illness, I was a very active person”. The other possible answers were “Never true,” “Rarely true,” and “Sometimes true.” Statistical significance between the two groups: ^∗^*p* < 0.05.

Considering professional activity, 89% of fibromyalgia patients (versus 67% of migraine patients, *p* < 0.05) considered to have been very active people before illness (Figure 5).

## 4. Discussion

This quantitative and comparative study showed that despite a comparable level of pain score and invalidating impact of the disease in the two groups (fibromyalgia and migraine), the prevalence of abuses and deprivations (during infancy) reported by fibromyalgia women, as well as their current subjective sensitivity to stress and stimuli, was higher than in the migraine group. In addition, the fibromyalgia patients considered themselves to have been hyperactive women before their illness. Anxiety and depression were also significantly more frequent than in migraine patients.

In our study, physical abuses in childhood were retrospectively reported by 25% of adult fibromyalgia women. This prevalence was significantly higher than that measured in the migraine group or for the general female population [17]. Even if the causes of fibromyalgia are currently unknown, several studies suggested that physical traumas occurring during childhood could contribute to the physiopathology of this syndrome [7, 20, 21]. This etiology would differ from that of migraine: while the genetic and neurovascular origin of migraine is frequently reported [22], the fibromyalgia syndrome rather would be associated with psychic and environmental events occurring along a traumatic life history. The psycho-affective impact of traumatic experiences would contribute to their illness.

Moreover, the fibromyalgia patients declared more affective deficiencies than did migraine women. A lack of attention or of parental presence was more frequent and could have durably affected these patients [23, 24]. Some recent studies showed that a premature birth, a maternal deprivation or a kind of insecure affection could be associated with chronic pain and foster the pain sensitivity (for review, cf. [21]). This was confirmed by the high “emotional neglect” score measured by CTQ in fibromyalgia patients. This score was also very high in migraine patients, and the difference between the two groups was not significant. This may seem inconsistent with the precedent differences mentioned for affective deprivation. However, this apparent inconsistency could be explained by the fact that whereas the CTQ referred to the relationships between the child and all the members of the family, concerning lack of affection, the child stated directly father and mother affection, so referred to the primordial attachment [23].

In addition to our observations, a recent study revealed that other childhood adversities could be associated with fibromyalgia: financial difficulties, conflicts in the family, parental divorce, chronic illnesses in the family, or alcohol problems [25]. These types of adversities could be at the origin of parental difficulties predisposing to a familial climate with lack of affection, emotional deprivation, or physical abuse of the patients during childhood.

The sensitivity to stimuli reported by patients and the results of the Holmes and Rahe test showed a multimodal hypersensitivity (cold, moisture, heat, full moon, and flavors), as well as increased sensitivity to stress, in fibromyalgia patients compared to those with migraine. Hypersensitivity in fibromyalgia has recently been shown to be correlated with neurophysiological events and depression [26].

Early traumatic events and affective deprivations during childhood could have disturbed the development of neurotransmitter systems, the pain processing, and the hypothalamic pituitary adrenal axis involved in stress management [7, 27, 28]. In fibromyalgia patients, early physical abuse could then have increased responsiveness of the central nervous system to a variety of stimuli. This central sensitization would occur because of decreased functional connectivity in the descending pain-modulating system [8] and augmented responses in sensory integration [26]. Moreover, modification of diurnal cortisol level associated with childhood maltreatment has been described in fibromyalgia and could contribute to great emotional distress and high catastrophism observed in these patients [29, 30]. Increased sympathetic activity has been suggested in fibromyalgia [31] and could explain the high sensitivity to stress.

Ergomania or professional hyperactivity was another feature observed in 89% of cases of fibromyalgia patients, as already described [32, 33]. Although in our study the declarative questionnaire could have been tainted by idealization, another study showed that the entourage of the patients usually confirmed this singularity [33]. Ergomania could be the expression of a low self-esteem associated with parental failures, or a strategy to escape depression as it has been described in maltreated children [32, 34].

Moreover, it should be noted that, in our study, depression was still observed in 57% of fibromyalgia patients (versus 9% of migraine patients, *p* < 0.001). The higher depressive rate of fibromyalgia patients could explain multimodal hypersensitivity [26] as well as their painful memory, catastrophism, and sleep alteration characteristic of the syndrome [35–37].

Finally, the nonrestorative awake sleep, associated with depression, anxiety, and multimodal hypersensitivity, could induce a permanent state of hypervigilance in fibromyalgia patients. Initially, this state would have been induced by insecure environment of fibromyalgia patients during childhood [21, 38]. All senses were maintained in alarm. Patients would then become more sensitive to stressful events (separation, layoff, financial problems, accident, disease, bereavement, etc.), with exacerbated physic or psychic stress related to daily life events [21, 30]. All these parameters associated with defensive hyperactivity and sleep deprivation would constitute a fertile field for the development of fibromyalgia. With time, sleep deprivation, natural decline of performances, and resistance with age would amplify symptoms. Hypervigilance and hyperactivity would result in overactivity, depression, and chronic fatigue, which ultimately lead to state of breakdown and exhaustion or “burn out” (professional, parental, conjugal, domestic, and social). The occurrence of musculoskeletal diseases such as osteoarthritis, hypothyroidism, inflammatory rheumatism, or other painful diseases would switch the patient from ergomania to invalidity. This would generate misunderstanding from relatives and medical team. Only personal positive adaptive and coping strategies could delay the switchover [39]. Patients with chronic migraine are frequently affected by diffuse pain, framed in fibromyalgia diagnosis. This comorbidity could be supported by common pathophysiological mechanisms [40].

This study has several strengths including the relatively large sample with clear diagnoses and the use of validated psychometric tests in order to objectivize psycho-affective parameters. However, it also includes some limitations. Bias is possible because it is a retrospective study based on self-reports. Patient memories may not be accurate and not objective. Moreover, the recruitment from the same unit and, a fortiori, from the same region and country, may limit generalizability of the findings. Results need to be confirmed by a multicenter international study. Further studies might also examine the role of emotional neglect in migraine patients.

Nevertheless, the results corroborate and objectivize our clinical experience and conform with the existing literature. The study invites to more attention to psycho-affective aspects in the treatment of fibromyalgia patients.

## 5. Conclusion

Our results confirm that life history and sensitivities of fibromyalgia patients should be more systematically taken into consideration in clinical practice. Fibromyalgia patients considered themselves as being more sensitive mentally and physically compared to migraine patients. They also have higher depression levels. Treatment of fibromyalgia syndrome taking account of psycho-affective impact of life experiences, stress management, behavioral, and coping strategies, should limit further examinations, medical nomadism, and stigmatization of fibromyalgia patients.
